# Copper stress response in yeast *Rhodotorula mucilaginosa* AN5 isolated from sea ice, Antarctic

**DOI:** 10.1002/mbo3.657

**Published:** 2018-06-21

**Authors:** Guangfeng Kan, Xiaofei Wang, Jie Jiang, Chengsheng Zhang, Minglei Chi, Yun Ju, Cuijuan Shi

**Affiliations:** ^1^ School of Marine Science and Technology Harbin Institute of Technology at Weihai Weihai China; ^2^ Tobacco Integrated Pest Management of China Tobacco Tobacco Research Institute of Chinese Academy of Agricultural Science Qingdao China

**Keywords:** adaptive responses, Antarctica yeast, copper stress, proteomics

## Abstract

Heavy metal pollution in Antarctic is serious by anthropogenic emissions and atmospheric transport. To dissect the heavy metal adaptation mechanisms of sea‐ice organisms, a basidiomycetous yeast strain AN5 was isolated and its cellular changes were analyzed. Morphological, physiological, and biochemical characterization indicated that this yeast strain belonged to *Rhodotorula mucilaginosa* AN5. Heavy metal resistance pattern of Cd > Pb = Mn > Cu > Cr > Hg was observed. Scanning electron microscopic (SEM) results exhibited altered cell surface morphology under the influence of copper metal compared to that with control. The determination of physiological and biochemical changes manifested that progressive copper treatment significantly increased antioxidative reagents content and enzymes activity in the red yeast, which quench the active oxygen species to maintain the intercellular balance of redox state and ensure the cellular fission and growth. Comparative proteomic analysis revealed that, under 2 mM copper stress, 95 protein spots were tested reproducible changes of at least 10‐fold in cells. Among 95 protein spots, 43 were elevated and 52 were decreased synthesis. After MALDI TOF MS/MS analysis, 51 differentially expressed proteins were identified successfully and classified into six functional groups, including carbohydrate and energy metabolism, nucleotide and protein metabolism, protein folding, antioxidant system, signaling, and unknown function proteins. Function analysis indicated that carbohydrate and energy metabolism‐, nucleotide and protein metabolism‐, and protein folding‐related proteins played central role to the heavy metal resistance of Antarctic yeast. Generally, the results revealed that the yeast has a great capability to cope with heavy metal stress and activate the physiological and protein mechanisms, which allow more efficient recovery after copper stress. Our studies increase understanding of the molecular resistance mechanism of polar yeast to heavy metal, which will be benefitted for the sea‐ice isolates to be a potential candidate for bioremediation of metal‐contaminated environments.

## 1 INTRODUCTION

Copper (Cu) is an indispensable micronutrient and act as key roles as catalytic cofactor in cellular redox reactions and metal homeostasis (Burkhead, Reynolds, Abdel‐Ghany, Cohu, & Pilon, 2009). Presently, copper is extensively applied in industry and agriculture, resulting in serious copper pollution in environments (Martins, Hanana, Blumwald, & Gerós, 2012; Zou et al., 2015). Cu is very reactive in free form and excess Cu is strongly biotoxic to cells by oxidative stress, which can cause the damage of proteins, lipids, and nucleic acids (Ravet & Pilon, 2013; Sáez, Roncarati, Moenne, Moody, & Brown, 2015). Accordingly, understanding the metabolism response of microorganisms induced by heavy metals, including copper, is nowadays the dominating goal of scientific research on metal detection and removal.

The surroundings of microorganisms encased in Antarctic sea‐ice matrix are low temperatures and high light levels, with the only liquid being pockets of concentrated brines (Thomas & Dieckmann, 2002). Antarctica is often considered as one of the last pristine regions, but some studies have reported that there is a tendency to increase the heavy metal contaminants, such as As, Cd, Cr, Cu, Hg, Ni, Pb, and Zn, due to atmospheric circulation and anthropogenic contamination from modern industries (Planchon et al., 2002; Trevizani et al., 2016; Yin et al., 2006), and the heavy metals content is even higher in organisms of the Southern Ocean than those from other oceans (Bargagli, 2000).

Presently, the study highlight of Antarctic heavy metals was focused on their attribution in different sites of snow, ice, and sediments (Ferrari et al., 2001; Planchon et al., 2002; Yin et al., 2006), and different biota including bacteria, algae, filter‐feeders, invertebrates, and vertebrates (de Moreno, Gerpe, Moreno, & Vodopivez, 1997; De Souza, Nair, Loka Bharathi, & Chandramohan, 2006; Runcie, Townsend, & Seen, 2009; Yin et al., 2006). De Souza et al. (2006) found that about 29% and 16% bacterial isolates from Antarctic sea water were resistant to 100 ppm of Cd and Cr respectively, among which mostly were pigmented strains. The Antarctic limpet *Nacella concinna* under higher concentrations of heavy metals (Fe, Al, and Zn) was associated with higher superoxide dismutase (SOD) and catalase (CAT) activity to maintain tissue redox ratio balance (Weihe, Kriews, & Abele, 2010). Duquesne, Riddle, Schulz, and Liess (2000) found that the LC_50_ values of Antarctic gammarid *Paramorea walkeri* were 970 μg/L for Cu and 670 μg/L for Cd. The characteristics of bioaccumulation demonstrated that *Paramorea walkeri* was a biological indicator to monitor heavy metal contamination in Antarctic environment.

Yeasts are unicellular organisms with strong exterior cell wall, and usually can survive in many kinds of adversity. Researchers have isolated and characterized diverse metal‐resistant yeasts, such as *Candida*,* Rhodotorula*,* Aureobasidium*,* Cryptococcus*,* Saccharomyces*,* Hansenula*,* Kluyveromyces*,* Zygosaccharomyces*,* Pichia*,* Trichosporon*,* Debaryomyces*,* Yarrowia*, and *Schizosaccharomyces*. However, there is little information on the combined responses of Antarctic yeasts to heavy metal. In this study, a cold‐active yeast strain was identified by sequencing of the 26S rDNA as *Rhodotorula mucilaginosa* strain NA5. It is the first report demonstrating high tolerance toward heavy metals and morphological and physiological changes of the yeast cells induced by copper. This work provides information regarding ecological response of cold‐active yeast under heavy metal conditions and also lays foundation for bioremediation to remediate the heavy metal‐contaminated areas.

## 2 MATERIALS AND METHODS

### 2.1 Microorganism and culture

The yeast strain AN5 was isolated from Antarctic sea ice collected by the 23rd China Antarctic scientific expedition. Melting sea ice was progressively diluted and spread on 2216E agar plate at 10°C. After 7 days culture, many bacteria and yeasts were obtained by single colony isolation. Yeast AN5 was cultured at 10°C on YEPD medium (10.0 g of yeast extract, 20.0 g of peptone, and 20.0 g of dextrose, in 1,000 ml of sterilized sea water) at speed of 120 r/min. Additional CuSO_4_ (final concentration 2 mM) was added as copper stress.

To determine the optimal growth temperature, AN5 was spread on agar YEPD medium. Cell color and growth status were observed after 7 days culture at 4, 10, 20, and 30°C, respectively.

### 2.2 Morphological characteristics

The preliminary morphological analysis included the colony morphology and cell morphology referred to Kurtzman, Fell, Boekhout, and Robert (2011). The yeast cells were fixed on small glass pieces and dehydrated with 10, 20, 30, 50, 70, and 90% ethanol solutions sequentially. The cell morphology was observed with a scanning electron microscope (SEM) (Hitachi, S‐3400N Japan), and the acceleration voltage was constant at 5 kV.

### 2.3 Phylogenetic analysis

The yeast 26S rDNA D1/D2 region amplification was used for molecular identification. The primer pairs NL‐1 (5′‐GCATATCAATAAGCGGAGGAAAAG‐3′) and NL‐4 (5′‐GGTCCGTGTTTCAAGACGG‐3′) were used (Kurtzman & Robnett, 1997). The amplification was performed on a thermocycler (Life express, Bioer, China) referred to Kurtzman and Robnett (1997). The PCR product was electrophoresed on 1.0% agarose gel and sequenced by Shanghai Personal Biotechnology Limited Company (Shanghai, China).

For phylogenetic analyses, some *Rhodotorula* sequences were got from GenBank, and then edited using the program BioEdit V7.090 (Hall, 1999). The phylogenetic tree was constructed using the neighbor‐joining method in ClustalX program. Bootstrap analysis was performed from 1,000 random resamplings. The phylogenetic tree was displayed using Mega 6.0 written by Tamura, Stecher, Peterson, Filipski, and Kumar (2013).

### 2.4 MIC Determination of heavy metals

The minimal inhibitory concentration (MIC) of the metals for the strain was determined by the method as described by Rajpert, Skłodowska, and Matlakowska (2013). Resistance to metals was tested by growth in liquid YEPD medium supplemented with Cu^2+^, Cd^2+^, Pb^2+^, Cr^3+^, Mn^2+^, and Hg^2+^ at concentrations ranging from 10 mM to 1,200 mM. The flasks were incubated at 20°C for 6 days, and growth was monitored by OD_600_ measurement. The lowest concentration of metal inhibiting the visible growth of the microorganisms was considered as the MIC of the metal against the test strain.

### 2.5 Assay of biochemical indices

For the determination of enzyme activities, 100 mg fresh weight (FW) cells was homogenized in 20 ml 50 mM phosphate‐buffered saline (pH 7.8) using a prechilled mortar and pestle, then centrifuged at 12,000*g* for 30 min at 4°C. The supernatant was used for the determination of SOD, CAT, peroxidase (POD), glutathione reductase (GR) activities, and malondialdehyde (MDA), glutathione (GSH), and carotenoid content. For each biochemical indices assay, three independent biological replicates were sampled from each control and treatment.

SOD activity was determined according to the method of Chowdhury and Choudhuri (1985) based on the inhibition of the photochemical reduction of nitro blue tetrazolium (NBT). The measurement and calculation of CAT and POD activities were measured based on the method of Chance and Maehly (1955). The carotenoid content was assessed using multi‐parameter flow cytometry method documented by Freitas et al. (2014). GSH content was determined as described by Anderson (1985), and the MDA concentration was determined based on the methods of Heath and Packer (1968). The GR activity was determined using the methods of Pinto, Mata, and Lopezbarea (1984), and the GR activity was expressed as the decrease in OD_340_/(min•g FW).

### 2.6 Protein extract, two‐dimensional gel electrophoresis (2‐DE), and image analysis

Cells were collected by centrifugation at 4,000*g* for 8 min at 4°C and washed twice with precooling distilled water. The precipitation was frozen at once by liquid nitrogen and stored at −80°C for subsequent protein extraction. One gram wet yeast cells gathered equally from three different culture flasks were blended as a biological replicate. Proteins were extracted by the procedure of Wang et al. (2013) and Méchin, Damerval, and Zivy (2007). The protein concentrations were measured using the Bradford method (Bradford, 1976).

The isoelectrofocusing (IEF) was performed on the Bio‐Rad 2‐DE system as described by the manufacturer. After adding 400 μg proteins, dry Immobilized pH gradient (IPG) strips (pH 4.0–7.0, 24 cm linear) were rehydrated and focused in 450 μl rehydration buffer at 20°C using a continuous increase in voltage up to 10,000 V until reaching total 80,000 V•h. Focused IPG strips were immediately equilibrated for 2 × 20 min for protein reduction and alkylation by adding DTT (1%) and iodoacetamide (2.5%), separately. The second dimension SDS‐PAGE was carried out with 12% (w/v) polyacrylamide gels under the current of 25 mA, and the proteins were visualized by Commassie brilliant blue R‐250 staining. Differentially expressed proteins (DEPs) were spots which showed significant and reproducible changes of at least 10 folds. Protein spots detection, abundance determination, matching, and statistical analysis were carried out using the PDQuest 2‐D software 8.01 (Bio‐rad, USA).

### 2.7 Protein identification and data search

The DEP spots were destained and extracted following the protocol described by Sun et al. (2016). Protein spots were excised from the SDS‐PAGE gels and destained with 30% acetonitrile (ACN) containing 50 mM NH_4_HCO_3_. The lyophilized gel pieces were rehydrated in 25 μg/ml trypsin for overnight at 37°C. After digestion, the peptides were extracted with 2.5% trifloro acetic acid (TFA) in 50% ACN. Extracts were pooled and lyophilized. Peptide mass maps were obtained in positive ion reflector mode with 1,000 laser shots per spectrum by the 4,800 MALDI‐TOF/TOF MS Analyzer (Applied Biosystems, USA). Monoisotopic peak masses were automatically determined within the mass range 700–4,000 Da. The required peptide mass spectra were submitted to GPS explorer workstation, and then searched the NCBInr yeast protein database using MASCOT search engine (http://www.matrixscience.com). The parameters were as follows: taxonomic category, NCBInr yeast database (October 8, 2016); no MW/pI restrictions; trypsin cleavage, one missed cleavage allowed, carbamidomethylation set as fixed modification, oxidation of methionines allowed as variable modification, and fragment tolerance set to ±0.2 Da for MS and ±0.3 Da for MS/MS. Protein scores of greater than 72 were considered statistically significant (*p* < 0.05). Protein identifications were accepted if they contained at least two matched peptides. For protein spot 6508, only one matched peptide had also been accepted considering its protein score of 84 and confidence interval of 99.62%. In the search engine, proteins were successfully identified based on their scores of at least 95% confidence interval. Referring the searching results of Gene Ontology (GO) database (http://www.geneontology.org/) and Kyoto Encyclopedia of Genes and Genomes (KEGG) database (http://www.kegg.jp/), the identified proteins were classified into different biological functional groups.

### 2.8 Statistical analysis of data

Data were performed with at least three replicates and expressed as the mean value ± SD. Difference significance were analyzed by SPSS 13.0 (SPSS Inc., Chicago, USA). *p* < 0.05 was considered to be a statistical significant difference.

## 3 RESULTS

### 3.1 Identification of the yeast strain

**Figure 1:**
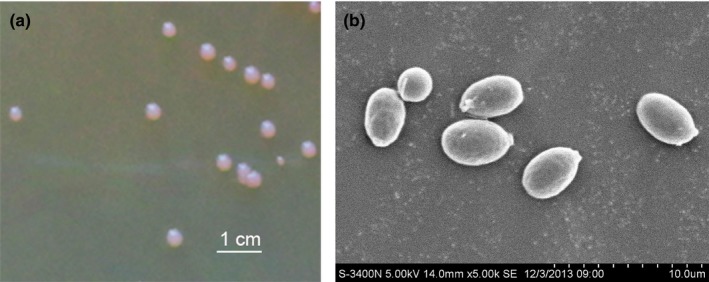
The colony morphology (a) and cell morphology (b) of AN5 grown on YEPD medium plate after 7 days growth

**Figure 2:**
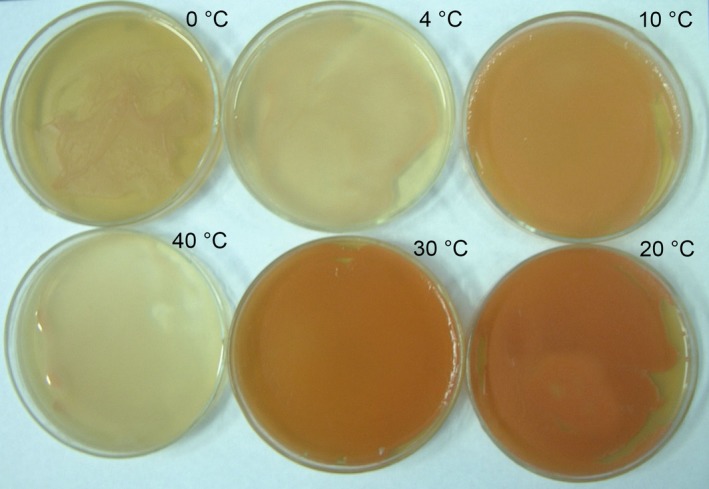
Growth of strain AN5 on YEPD medium plate under 0, 4, 10, 20, 30, and 40°C

After 7 days culture at 10°C, the colony of AN5 on YEPD plate was round, orange, and opaque with a regular edge, and measured about 4 mm across (Figure 1a). Cell was oval, and the asexual reproductive mode was a single‐ended budding (Figure 1b). In addition, the cell didn't produce ascospore and pseudohypha. The strain could grow from 0 to 40°C, and optimized growth temperature was 20°C (Figure 2).

**Figure 3:**
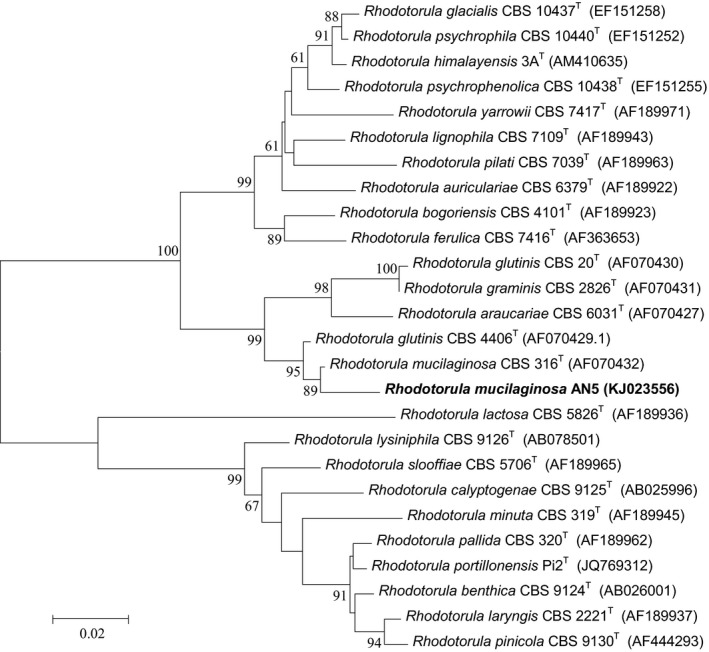
Phylogenetic tree of strain AN5 and related *Rhodotorula*, obtained by neighbor‐joining analysis of the D1/D2 region of the 26S rDNA. Percentage bootstrap values of 1000 replicates are given at each node. GenBank accession numbers are indicated after strain designation (names in boldface correspond to sequences determined in this study; T, type strain)

The AN5 D1/D2 region of 26S rDNA sequence was available in the NCBI GenBank database under the accession number of KJ023556. Nucleotide blast for the sequences in GenBank database revealed a 98% identity with species *Rhodotorula mucilaginosa*. The phylogenetic tree constructed by the neighbor‐joining method also showed that strain AN5 was close to *R. mucilaginosa* (Figure 3).

### 3.2 Heavy metals resistance analysis

**Figure 4:**
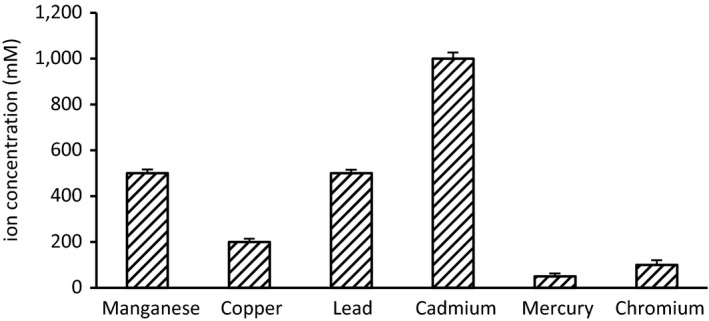
Heavy metals tolerances of strain AN5. Metals resistance, expression with the minimal inhibitory concentration, was tested by growth in liquid YEPD medium with 10 mM to 1,200 mM of Cu^2+^, Cd^2+^, Pb^2+^, Cr^3+^, Mn^2+^, and Hg^2+^, separately. Cultures were performed at 20°C, 120 r/min for 6 days, and growth was monitored by OD_600_ measurement

The *R. mucilaginosa* AN5 was tested for its resistance against Cu^2+^, Cd^2+^, Hg^2+^, Mn^2+^, Cr^3+^, and Pb^2+^. Three biological replicates were carried out per metal. The results showed that the yeast strain was tolerant to different concentrations of heavy metals (Figure 4). A heavy metal resistance pattern of Cd > Pb = Mn > Cu >Cr > Hg was observed with a maximum MIC for 1,000 mM Cd and a minimum for Hg (50 mM) (Supporting Information Figure S1).

### 3.3 Morphological changes under copper stress

**Figure 5:**
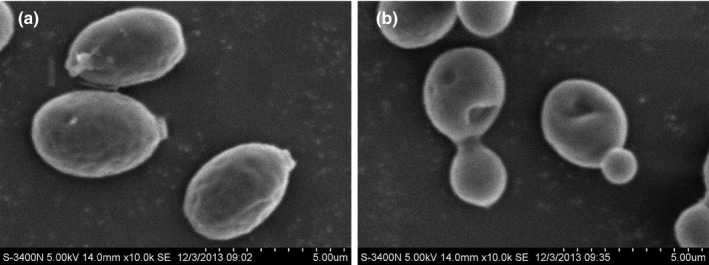
Scanning electron microscopic pictures of AN5 grown in the presence of copper ions at a concentration of 2 mmol/L. (a) Control; (b) Copper ion stress

The results of SEM displayed that some changes occurred in the cell surface morphology with copper stress (Figure 5). The surface of the control yeast appeared as smooth, whereas the yeast cells grown at 2 mM of Cu^2+^ were depression and shrunken, and multiple pores were observed on the surface. The cell size reduced under metal stress from 5 × 3 μm to 3.5 × 3 μm. Similar changes were also observed in psychrotolerant yeast *Cryptococcus* sp. upon exposed Zn, Cu, Pb, and Cd (Singh, Raghukumar, Parvatkar, & Mascarenhas‐Pereira, 2013).

### 3.4 Biochemical changes under copper stress

**Figure 6:**
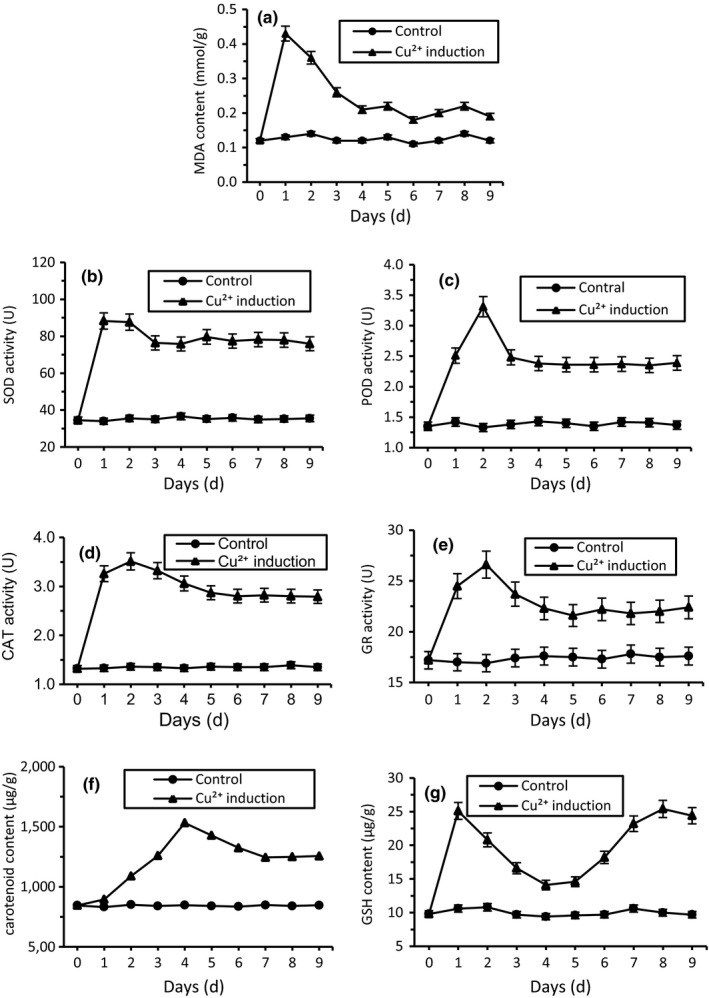
Biochemical changes of *R. mucilaginosa* AN5 exposed to 2 mM copper stress. (a) MDA; (b) SOD; (c) POD; (d) CAT; (e) GR; (f) Carotenoid; (g) GSH

MDA is an indicator of stress damage, producing during the membrane lipids peroxidation (Ohkawa, Ohishi, & Yagi, 1979). Figure 6a showed that little change in MDA content was observed in untreated yeast cells during the whole exposure period. At 2 mM Cu^2+^ treatment, MDA content was rapidly accumulated and reached a maximum of 0.43 mmol/g FW at day 1, and then downregulated swiftly to 0.21 mmol/g FW at day 8 but still significantly higher than the control group (*p* > 0.01). These findings demonstrated that a high rate of lipid peroxidation and loss of cell membrane integrity occurred in cells inoculated with copper ion.

Figure 6b–e showed that the change trends of four antioxidant enzymes activity (SOD, POD, CAT, and GR) were similar. Throughout the experiment, these 4 enzymes remained flat at all time without the addition of Cu^2+^. Exposed to 2 mM Cu^2+^, SOD, POD, CAT, and GR activity began to rise rapidly and got to utmost value of 88.3, 3.31, 3.51, and 26.6 U/(min g FW) at day 1 or 2, respectively. In the following days, the activity of antioxidant enzymes slightly decreased, but was still beyond the control group obviously over time (*p* < 0.01).

The changes of carotenoid and GSH content in yeast cells (Figure 6f,g) without treatment remained unchanged during 10 days culture phase, which were similar to antioxidant enzymes. At 2 mM Cu stress, carotenoid was induced gradually to the maximum of 1,532 μg/g FW at day 4. Then the pigment content decreased slowly, but still significantly higher than the control group. Cu^2+^ stress also induced the rapid accumulation of GSH, and the concentration of GSH reached its maximum value 25.1 μg/g FW only after 1 day of treatment. After 3 days gradual drop, GSH content ascended slowly again from day 5 and reached the same level to maximum. In the whole experiment period, the GSH content of treated group was higher than in the control group.

The instant increase in both enzymatic and nonenzymatic defense substances was coincident with the decreases in MDA level (Figure 6a), further indicating that the higher antioxidants in cells is responsible for stress tolerance.

### 3.5 Protein profiles under copper stress

**Figure 7:**
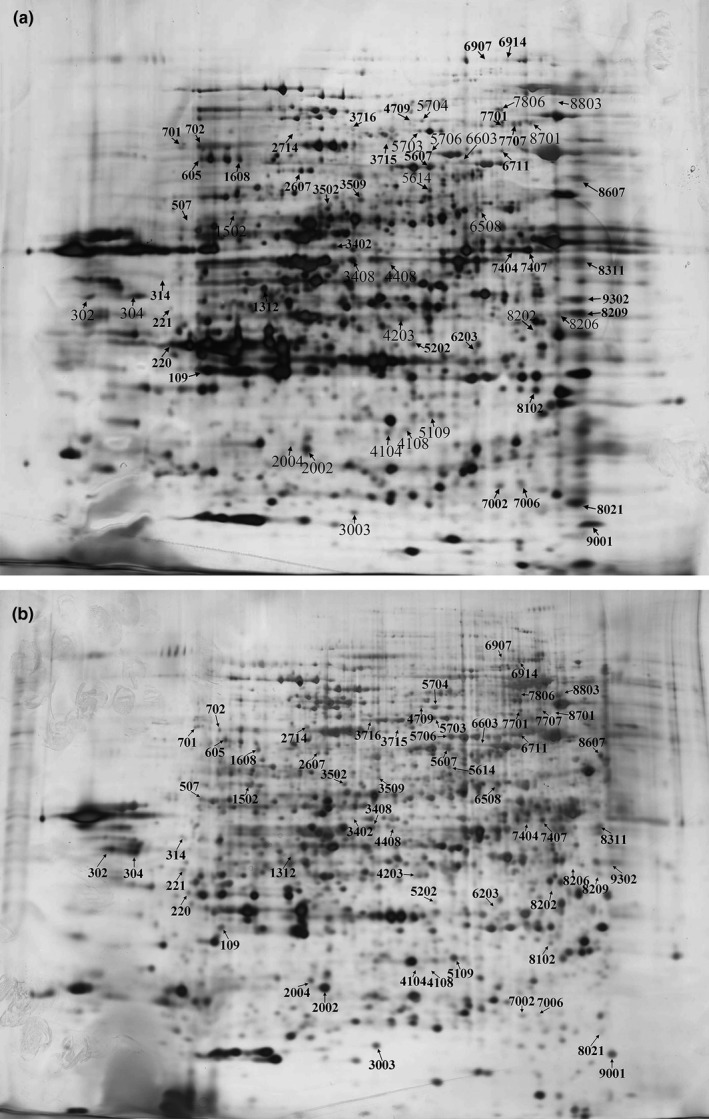
Representative 2‐DE gel images of *R. mucilaginosa*. (a and b) represents 2D gels from samples treated with 0 and 2 mM CuSO_4_, respectively. Arrows indicate reproducible and significant (*p* < 0.05) protein changes at least 10 folds after Cu^2+^ treatment when compared to control

Proteins from control and copper‐treated yeast cells were separated using 2‐DE, and a representative experiment is shown in Figure 7. Three biological replicates were analyzed and twofold threshold limit was set. More than 2,183 protein spots were automatically detected on each gel with PDQuest 2‐D software 8.01 from six gels, and followed by manual editing. The image analysis showed that 988 protein spots were changed significantly in abundance, among which 511 protein spots were upregulated and 477 protein spots were downregulated in the copper‐treated cells compared to the control cells.

**Figure 8:**
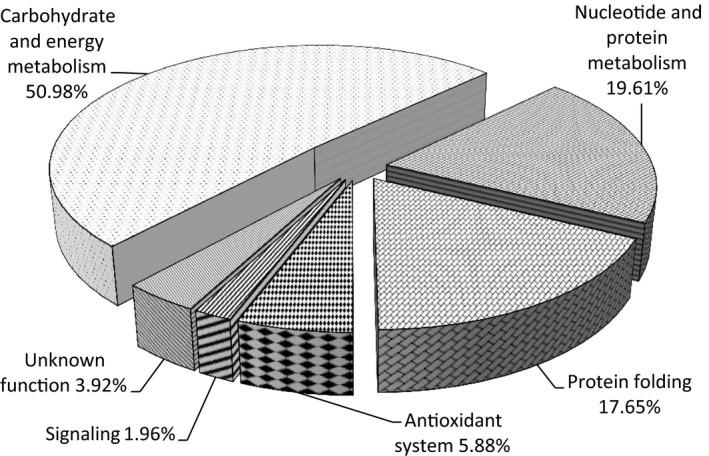
Pie chart showing functional categories of identified proteins

A total of 95 DEP spots (more than 10‐fold changes) were sent for protein identificaion using MALDI‐TOF/TOF MS analysis, and 51 spots were successfully identified based on NCBI‐yeast databases search. These proteins information was listed in Table 1. Some proteins were found to belong to the same, possibly because of different isoforms or posttranslational modifications. According to GO, KEGG database and literature searches, 51 proteins were categorized into six functional groups: carbohydrate and energy metabolism (26, 50.98%), nucleotide and protein metabolism (10, 19.61%), protein folding (9, 17.65%), antioxidant system (3, 5.88%), signaling (1, 1.96%), and unknown function proteins (2, 3.92%), as shown in Figure 8. The results suggested that carbohydrate and energy metabolism, nucleotide and amino acid metabolism, and protein folding were disrupted and played key roles in protecting yeast cells from damage upon copper exposure.

**Table 1:** Differentially expressed proteins identified in control and Cu‐treated *R. mucilaginosa* cells

Spot no.^a^	Protein name	Accession no. (gi)	Predicted Mr/pI	Observed Mr/pI	Number of matched peptides	Reference species	Protein score	Protein score C.I.%	Fold change b	*p*‐value
*Carbohydrate and energy metabolism*										
109	L‐malate dehydrogenase, mitochondrial	472581873	35.58/8.76	34.27/6.58	5	*Rhodosporidium toruloides*	77	98.17	0.086	0.01
220	L‐malate dehydrogenase, mitochondrial	472581873	35.58/8.76	37.06/6.87	5	*Rhodosporidium toruloides*	205	100.00	0.056	0.00
221	Alcohol dehydrogenase	472587838	38.20/6.46	42.26/6.87	5	*Rhodosporidium toruloides*	109	100.00	+	0.01
605	6‐Phosphogluconate dehydrogenase	472587565	52.44/6.21	58.32/6.73	10	*Rhodosporidium toruloides*	98	99.98	0.086	0.00
701	NADH dehydrogenase (ubiquinone) flavoprotein 1	472584566	56.55/8.16	62.03/6.88	12	*Rhodosporidium toruloides*	242	100.00	0.083	0.01
702	6‐Phosphogluconate dehydrogenase	472587565	52.44/6.21	62.06/6.77	9	*Rhodosporidium toruloides*	85	99.74	0.080	0.01
1502	2‐Methylcitrate synthase	472580577	21.99/8.67	48.67/6.62	3	*Rhodosporidium toruloides*	73	95.23	10.65	0.02
3402	Pyruvate dehydrogenase E1 component subunit alpha	472583762	47.58/8.09	46.23/6.18	9	*Rhodosporidium toruloides*	264	100.00	0.092	0.00
3408	Pyruvate dehydrogenase E1 component subunit alpha	472583762	47.58/8.09	45.29/6.16	9	*Rhodosporidium toruloides*	172	100.00	12.38	0.01
3502	Dihydrolipoyl dehydrogenase	472585468	52.44/6.81	55.68/6.14	13	*Rhodosporidium toruloides*	245	100.00	0.096	0.01
3715	NADH dehydrogenase (ubiquinone) Fe‐S protein 1	472583921	81.76/7.21	76.31/5.66	15	*Rhodosporidium toruloides*	190	100.00	+	0.02
4104	Phosphoglycerate mutase 1	472585349	24.39/6.68	26.31/5.76	6	*Rhodosporidium toruloides*	178	100.00	‐	0.03
4408	3‐Deoxy‐8‐phosphoheptulonate synthase	472582537	40.00/6.42	45.37/5.63	4	*Rhodosporidium toruloides*	77	98.23	11.34	0.01
4709	Oxoglutarate dehydrogenase	342320257	127.33/6.01	94.28/5.46	8	*Rhodotorula glutinis*	149	100.00	+	0.02
5607	UDP‐galactopyranose mutase	472586186	56.29/6.05	63.22/5.42	4	*Rhodosporidium toruloides*	79	98.76	0.026	0.00
6508	Enolase	501310447	46.36/5.14	50.10/5.12	1	*Pseudozyma hubeiensis*	84	99.62	12.65	0.01
5706	Pyruvate kinase	472583934	56.42/6.30	60.15/5.23	6	*Rhodosporidium toruloides*	169	100.00	13.15	0.01
6603	2‐Isopropylmalate synthase	342320438	67.90/5.89	59.18/5.18	8	*Rhodotorula glutinis*	175	100.00	‐	0.01
6711	Pyruvate dehydrogenase E2 component	472582629	61.32/6.52	61.85/5.02	12	*Rhodosporidium toruloides*	139	100.00	+	0.00
6914	Glycerol‐3‐phosphate dehydrogenase	342318964	147.96/8.64	129.87/5.21	12	*Rhodotorula glutinis*	128	100.00	+	0.03
7404	Enolase	1022840960	47.78/5.33	46.58/5.21	4	*Rhodosporidium toruloides*	77	98.25	0.090	0.00
7806	Glycogen phosphorylase	517243372	92.34/5.80	86.43/5.26	18	*Burkholderia sp*.	75	97.23	35.05	0.01
8102	Transaldolase	472586368	35.80/5.67	34.57/4.92	6	*Rhodosporidium toruloides*	142	100.00	0.078	0.01
8202	6‐Phosphogluconate dehydrogenase	472587565	52.44/6.21	45.13/4.96	9	*Rhodosporidium toruloides*	77	98.25	‐	0.01
8209	Pyruvate dehydrogenase E1 component subunit beta	472585974	42.92/5.74	46.19/4.62	4	*Rhodosporidium toruloides*	87	99.84	0.070	0.02
9302	Pyruvate dehydrogenase E1 component subunit beta	472585974	42.92/5.74	47.08/4.63	6	*Rhodosporidium toruloides*	70	90.18	0.086	0.00
*Protein folding*										
2714	Heat shock protein 70, hsp70A2	172718	70.50/5.05	69.82/5.89	11	*Saccharomyces cerevisiae*	269	100.00	+	0.01
3003	NB‐ARC and TPR repeat‐containing protein	298714756	164.84/5.44	20.58/5.88	6	*Ectocarpus siliculosus*	73	95.90	14.31	0.01
3716	Heat shock protein/chaperonin	472585379	89.72/6.11	85.63/6.14	8	*Rhodosporidium toruloides*	106	100.00	+	0.03
5703	T‐complex protein 1 subunit beta	472580721	56.46/5.70	70.46/5.42	12	*Rhodosporidium toruloides*	99	99.99	12.34	0.00
7006	70 kDa heat shock protein	171728	69.61/5.03	24.88/4.89	9	*Saccharomyces cerevisiae*	113	100.00	0.054	0.01
7407	Heat shock protein 70	562977171	70.19/5.03	42.73/4.85	8	*Ogataea parapolymorpha*	232	100.00	0.082	0.03
7701	Molecular chaperone DnaK	472580771	70.79/5.96	80.21/5.23	10	*Rhodosporidium toruloides*	322	100.00	0.066	0.02
7707	Heat shock protein 70A2	172718	70.50/5.05	80.23/5.11	12	*Saccharomyces cerevisiae*	217	100.00	0.026	0.02
8311	Heat shock protein 70	1708305	70.09/5.11	40.22/4.61	10	*Ogataea angusta*	124	100.00	0.054	0.01
*Nucleotide and protein metabolism*										
302	Elongation factor EF‐1 alpha subunit	472587418	50.17/9.15	47.21/6.98	6	*Rhodosporidium toruloides*	77	98.29	23.23	0.00
304	Elongation factor EF‐1 alpha subunit	472587418	50.17/9.15	48.09/6.84	9	*Rhodosporidium toruloides*	162	100.00	‐	0.02
314	ATP‐dependent RNA helicase RhlE	491634597	51.03/9.90	52.03/6.80	8	*Pseudoalteromonas luteoviolacea*	77	98.29	+	0.01
507	Elongation factor EF‐1 alpha subunit	472587418	50.17/9.15	48.92/6.43	9	*Rhodosporidium toruloides*	113	100.00	+	0.01
3509	IMP dehydrogenase	472580737	56.86/6.44	55.63/5.89	3	*Rhodosporidium toruloides*	104	100.00	0.052	0.03
5614	Elongation factor EF‐1 alpha subunit	472587418	50.17/9.15	56.28/5.38	6	*Rhodosporidium toruloides*	73	95.51	15.43	0.02
6203	Translation initiation factor eIF‐3 subunit 2	472585701	38.44/5.51	37.28/5.21	4	*Rhodosporidium toruloides*	105	100.00	0.086	0.01
7002	Putative chromatin remodeling complex atpase chain isw1 protein	485924588	127.25/5.99	24.38/5.21	8	*Neofusicoccum parvum*	74	96.27	0.084	0.01
8701	SARP family transcriptional regulator	517739234	123.61/5.86	79.63/4.89	13	*Salinispora arenicola*	73	95.71	‐	0.00
8803	Single‐stranded DNA binding protein	472580369	55.95/5.03	101.23/5.01	7	*Rhodosporidium toruloides*	88	99.86	52.56	0.03
*Antioxidative system*										
2002	Mitochondrial peroxiredoxin 6, 1‐Cys peroxiredoxin	472586343	24.95/5.86	23.43/6.02	4	*Rhodosporidium toruloide*	74	96.27	12.81	0.01
4108	Catalase A	472584117	87.82/6.21	25.68/5.42	7	*Rhodosporidium toruloides*	133	100.00	‐	0.02
5109	Mitochondrial peroxiredoxin 6, 1‐Cys peroxiredoxin	472586343	24.95/5.86	26.48/5.39	4	*Rhodosporidium toruloides*	152	100.00	12.88	0.01
*Signaling*										
5202	3‐beta hydroxysteroid dehydrogenase/isomerase	472581852	37.06/5.81	37.23/5.41	7	*Rhodosporidium toruloides*	91	99.93	0.080	0.02
*Unknown function*										
8607	Hypothetical protein GUITHDRAFT_161407	428182889	49.42/6.54	54.21/4.43	16	*Guillardia theta*	80	99.00	0.090	0.01
2004	hypothetical protein HMPREF1120_08112	378733681	185.26/5.12	25.24/6.02	7	*Exophiala dermatitidis*	74	96.27	21.46	0.01

a Spot number corresponds to the 2‐DE imagines in Figure 9a and b.

b Fold change is the ratio between average volume of each protein spots in Cu‐treated cells compared control cells. If the ratio is higher than 10‐fold, it is accepted as upregulation; if it is lower than 0.1‐fold, it is accepted as downregulation. “+/−” means that the protein was appeared/disappeared in copper‐induced cells.

## 4 DISCUSSION

Some metal ions are indispensable for numerous metabolic processes, but be toxic when present excess in concentration (Hall, 2002). Excess heavy metals can usually damage the lipids, proteins, and DNA, generally leading to cell death in yeast (Halliwell & Gutteridge, 1984; Morano, Grant, & Moyerowley, 2012). Also, heavy metals cause a significant threat to public health because of the accumulation in body throughout the food chains (de Moreno et al., 1997; Goutte et al., 2015). The purpose of this study was to discover the acclimation responses and adaption mechanism of Antarctic yeast *R. mucilaginosa* to heavy metal Cu. Therefore, the analysis of morphological, physiological, and proteomic changes of polar sea‐ice yeast AN5 exposed to Cu were carried out.

In this study, yeast NA5 was isolated from a sea‐ice sample with high heavy metals concentration (Lannuzel, Bowie, Merwe, Townsend, & Schoemann, 2011), and exhibited considerable growth in the presence of heavy metals, Cu^2+^, Cd^2+^, Hg^2+^, Mn^2+^, Cr^3+^, and Pb^2+^. The MIC of the heavy metals ranged from 50 to 1,000 mM, which was obviously higher than yeast *R. mucilaginosa* RCL‐11 (Villegas, Amoroso, & de Figueroa, 2005). The isolate was capable of growing in the presence of CuSO_4_ up to 200 mM, had unusually high levels of resistance to Cu as compared to the yeast species where *R. mucilaginosa* strain LM9 could grow in the medium containing 10 mM CuSO_4_ (Rajpert et al., 2013). In addition, Pb MIC of yeast AN5 was 500 mM, and higher than *R. mucilaginosa* 2S4 isolated from heavy metal‐contaminated sludge (Muñoz et al., 2012). A similar result was observed by Møller et al. (2011) who isolated bacterium proteobacteria, Firmicutes, Actinobacteria, and Bacteriodetes from Arctic sea‐ice brine and found that isolate was resistant to mercury. One of key reasons of high metal tolerance as compared to other yeasts may be the long adaptive modifications in Antarctic extreme surroundings including metal contamination. The variation in the metal tolerance was may be due to the presence of one or more types of tolerance strategies or resistance mechanisms exhibited by different organisms (Zafar, Aqil, & Ahmad, 2007).

Copper, like other heavy metals, also affects on the morphology and growth rate of the yeast cells. Under metal stress, the cell size reduced and cell wall shrunken, which was coincided with those reported by Singh et al. (2013) and Muneer, Lali, Iqbal, Shakoori, and Shakoori (2016). A prolonged lag phase was observed when yeast AN5 was grown in the presence of Cu^2+^. This delay in growth may be due to the consumption of metabolic energy to cope with metal stress (Muneer et al., 2016). However, cell overall growth was not much influenced completely. The studies of Chen et al. (2000) have shown that secretion of extracellular polymeric substance modified cell surface morphology during biosorption of Zn and Cu in *Desulfovibrio desulfuricans*.

Organisms surviving in heavy metal conditions require a complex suite of biochemical and metabolic adaptations. Heavy metals induce the intracellular overproduction of reactive oxygen species (ROS), including superoxide anion (O_2_∙^−^), hydroxyl radical (∙OH), and hydrogen peroxide (H_2_O_2_) (Nargund, Avery, & Houghton, 2008; Shanmuganathan, Avery, Willetts, & Houghton, 2004). To alleviate the lipid peroxidation from ROS, organisms have evolved enzymatic antioxidants, such as SOD, POD, CAT, and GR, and also possess nonenzymatic antioxidants that include reduced GSH and carotenoid (Moore, Breedveld, & Autor, 1989). ROS and antioxidant substances are often taken as a kind of balancing system, and if the balance is broken, the toxicity of Cu^2+^ in cells is apparent (Jiang et al., 2011). In the study, SOD activity got to the maximum on the first day under Cu‐pretreated stress, which indicated that the rapid increase in SOD might remove or decrease O_2_∙^−^ anion to produce H_2_O_2_. Subsequently, H_2_O_2_ and ∙OH were reduced or eliminated by the catalysis of CAT and POD. The results were similar to the antioxidant enzymes response under Cu^2+^ stress to other yeasts (Irazusta, Nieto‐Peñalver, Cabral, Amoroso, & Figueroa, 2013; Ribeiro et al., 2015). Some nonenzymatic system such as carotenoid and GSH directly react with ROS and scavenge them (Smirnoff, 1993). This study showed that induced carotenoid scavenged free radicals and ROS generated by heavy metal to avoid the oxidative damage. The study of Bhosale and Gadre (2001) also found all of 14 divalent cation salts studied resulted in a higher production of carotenoids.

To determine the protein alterations in response to heavy metal stress in polar yeast, a proteomic analysis by the method of 2‐DE and MALDI‐TOF/TOF MS was performed. To the best of our knowledge, proteomic analysis from the Antarctic organisms aimed at archaeon (Liao et al., 2016), bacteria (Kulkarni, Swamy, & Jagannadham, 2015), alga (Park, Jin, & Lee, 2008), and animals (Piechnik, Höckner, de Souza, Donatti, & Tomanek, 2017), and no study of proteome responses induced by heavy metal in Antarctic yeast was reported. Here, results of proteomics researches indicated that 51 DEPs were identified in Antarctic yeast *R. mucilaginosa* AN5, and they were classified into 6 categories by functional analysis, including carbohydrate and energy metabolism, nucleotide and amino acid metabolism, protein folding, antioxidant system, signaling, and unknown function proteins.

Carbohydrate and energy metabolism acted as key attribution in response to heavy metal adaptation. Heavy metal ion influenced the glycolysis pathway obviously in polar yeast, and 26 carbohydrate and energy metabolism‐related proteins were found significant changes under Cu stress (Table 1). In this study, glycogen phosphorylase (spot 7806) abundance elevated to catalyze glycogen into glucose‐6‐phosphate. The new‐synthesized glycerol‐3‐phosphate dehydrogenase (spot 6914) increased the intracellular 3‐phosphate glyceraldehyde concentration under copper stress. Moreover, we detected higher abundance of enolase (spot 6508) and pyruvate kinase (spot 5706), which accelerated the production of pyruvate. The upregulation of these enzymes in abundance suggested that copper stress enhanced the carbohydrate catabolism by glycolysis pathway. However, our study indicated that pentose phosphate pathway of carbohydrate catabolism, the key pathway in the generation of reducing energy NADPH (Chiapello, Daghino, Martino, & Perotto, 2010), was decreased by the downregulated abundance of 6‐phosphogluconate dehydrogenase (spot 605, 702 and 8202) and transaldolase (spot 8102). A decrease in pentose phosphate pathway was also observed in brown algae *Sargassum fusiforme* stressed by copper due to the decrease in the abundance of a potential transketolase (Zou et al., 2015). These results indicated that carbohydrate was preferably utilized for glycolysis than pentose phosphate pathway under copper stress.

Pyruvate dehydrogenase is a complex of three enzymes (E1, E2, and E3) that converts pyruvate into acetyl‐CoA. Under copper induction, the complex abundance was adjusted intricately, the synthesis of pyruvate dehydrogenase (E1) (spot 3408) and dihydrolipoyl transacetylase (E2) (spot 6711) increased, but pyruvate dehydrogenase (E1) (spot 3402, 9209 and 9302) and dihydrolipoyl dehydrogenase (E3) (spot 3502) decreased.

Under copper stress, the activity of the TCA cycle pathway seemed to be up regulated in yeast because the abundance of 2‐methylcitrate synthase (spot 1502) and oxoglutarate dehydrogenase (spot 4709) were increased, as observed in Cu‐treated *Penicillium janthinellum* (Feng et al., 2017). Furthermore, Cu treatment induced the biosynthesis of Fe‐S protein (spot 3715) involved in electron transportation as active site cofactors of NADH dehydrogenase (ubiquinone) in TCA cycle. The new‐synthesized Fe‐S protein improved the energy production during TCA pathway and increased the resistance of yeast to copper stress.

Besides the glycolysis‐ and TCA pathway‐related enzymes, some other enzymes in carbohydrate metabolism played important role in heavy metal adaptive mechanisms. 3‐Deoxy‐8‐phosphoheptulonate synthase (spot 4408) is involved in the biosynthesis of 3‐deoxy‐D‐manno‐octulosonate which is an essential component of rhamnogalacturonan II, a structurally polysaccharide in cell wall (Delmas et al., 2008). Moreover, the UDP‐galactopyranose mutase (spot 5607) is a key enzyme in the biosynthesis of galactofuranose, an essential component of fungal cell walls (Tanner, Boechi, Andrew, & Sobrado, 2014). Thus, the two enzymes abundance changes regulated the integrity and signal recognition in cell wall. Polysaccharides are known to bind heavy metals, and the increased cell wall polysaccharides might improve the buffering capacity of the yeast cell to copper. The result was consistent with Irazusta's studies reporting that polysaccharide was involved in resistance to copper in yeast *Candida fukuyamaensis* (Irazusta, Michel, & de Figueroa, 2016). Copper ion stress deactivated 2‐isopropylmalate synthase (spot 6603) and decreased indirectly the biosynthesis of some amino acids, such as leucine, isoleucine, and valine. The abundance of alcohol dehydrogenase (spot 221) ascended under copper stress and accelerated the reduction of pyruvate to ethanol via acetaldehyde and regenerate NAD^+^ to supply energy for glycolysis (Denness et al., 2011). The upregulation of this enzyme synthesis has been reported in *Corynebacterium glutamicum* stimulated by HgCl_2_ (Fanous, Weiss, Görg, Jacob, & Parlar, 2008). Perhaps this enzyme could serve to reestablish an NADH–NAD^+^ balance after exposure to stress conditions or act as a chaperonin to stabilize other essential enzymes (An, Scopes, Rodriguez, Keshav, & Ingram, 1991). Interesting, in TCA pathway, L‐malate dehydrogenase (spot 109 and 220) abundance decreased and resulted in the accumulation of malate. As a binding mediator of metal ions, malate could increase detoxicity to Cu in polar yeast (Tesfaye & Samac, 2001).

In all, in the central carbon metabolism pathway, glycolysis and the TCA cycle were triggered and the pentose phosphate pathway was inhibited under copper stress. Based on the data of this investigation, the acceleration of carbohydrate decomposition by glycolysis and subsequent TCA cycle in polar yeast are involved in the production of ATP and meet energy provision of cellular contend to copper (Akram, 2014; Feng et al., 2017), which was summarized in Figure 9.

**Figure 9:**
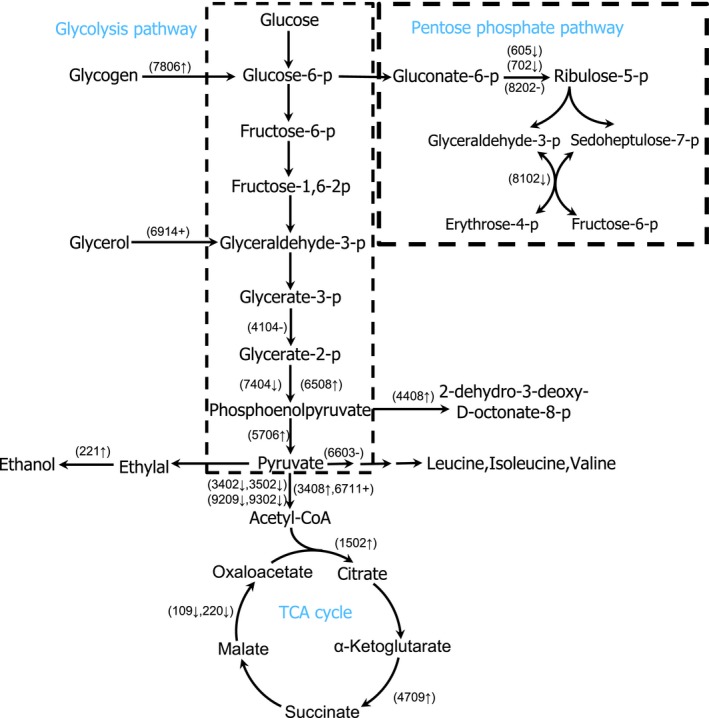
Metabolic adaptations suggested by the analysis of differential proteomic results. Proteins that changed abundance in response to copper stress are shown in spot number. ↑ denotes an increase, ↓ denotes a decrease, + denotes a appear, and – denotes a disappear in the abundance of protein isoforms

Heat shock proteins usually constitute 1%–2% of the total cellular proteins, which indicates their important functions in cells, including stabilization, proper folding, degradation, and translocation of proteins to maintain cellular homeostasis (Boshoff et al., 2004; Gupta, Sharma, Mishra, Mishra, & Chowdhuri, 2010; Irazusta et al., 2016). HSPs are extremely sensitive to even minor assaults and can be used as early stress signs (Gupta et al., 2010). Induction of heat shock proteins has been reported as defense mechanisms against a wide range of stress conditions, including heat shock, oxidative stress, UV radiation, and heavy metals, *etc*. (Parsell & Lindquist, 1993). Heavy metal usually induces synthesis of HSPs and chaperones, which can provide a protective function against metal toxicity. Our study detected some HSPs (heat shock protein 70 (spot 2714), NB‐ARC and TPR repeat‐containing protein (spot 3003), heat shock protein/chaperonin (spot 3716), and T‐complex protein 1 subunit beta (spot 5703)) were increased or de novo synthesized in polar yeast treated with copper ion. These enhanced HSPs prevented protein irreversible unfolding caused by ROS. Additionally, other HSPs, such as T‐complex protein 1, DnaK‐type molecular chaperon BiP, protein disulfide‐isomerase, HSP70, and mitochondrial chaperonin HSP60, were also found increased in organisms under heavy metal stress (Ahsan et al., 2007; Ireland et al., 2004; Sarry et al., 2006). Similarly, previous studies tested the relationship between copper oxidative stress and protection of HSPs in yeast *C. fukuyamaensis* (Irazusta et al., 2016) and *R. mucilaginosa* (Irazusta, Estévez, Amoroso, & de Figueroa, 2012). Thus, the abundance of HSPs and chaperone proteins were increased probably to stabilize protein conformation and function, and protect cells against heavy metal damages. However, heavy metal stress commonly causes the denaturation or dysfunction of proteins, most likely related with the presence of ROS processes, including HSP/chaperonin itself. Previous proteomic studies observed that HSP70 was significantly decreased in *Arabidopsis* exposed to cadmium stress (Sarry et al., 2006). In this study, HSP70 (spot 7006, 7407, 7707, and 8311) and molecular chaperone DnaK (spot 7701) were decreased by copper stress, which was consistent with the change of HSP70 in poplar stressed by cadmium (Kieffer, Dommes, Hoffmann, Hausman, & Renaut, 2008) and DnaK in *Anabaena* exposed with uranium (Panda, Basu, Acharya, Rajaram, & Apte, 2017). The downregulation of HSP/chaperone discovered that copper ion inhibited efficiently the chaperone‐assisted refolding of denatured proteins and destroyed partly the cellular homeostasis.

Heavy metal stress in yeast is known to increase nucleic acids and protein synthesis. Inosine‐5‐monophosphate (IMP) dehydrogenase play central role in purine nucleotide synthesis, catalyzing IMP to GMP, the precursor of the DNA and RNA. The upregulation of IMP dehydrogenase in this work supplied the components for DNA replication and transcription, and indirectly stimulated the protein biosynthesis, which is in line with the response of *Pseudomonas aeruginosa* exposed with chromium (VI) toxicity (Kılıç, Stensballe, Otzen, & Dönmez, 2010). The main function of single‐stranded DNA binding (SSB) protein is to bind to single‐stranded DNA and prevents annealing of single‐stranded DNA into double‐stranded DNA. Thus, the overexpression of SSB protein (spot 8803) is involved in DNA replication, recombination, and repair against the copper stress. ISW1‐type complexes can remodel the chromatin and adjust the structure of nucleosomal DNA. One of ISW1 complex functions is to repress the initiation of gene expression (Tsukiyama, Palmer, Landel, Shiloach, & Wu, 1999). Thus, in this study, the expression of chromatin remodeling complex ATPase isw1 protein (spot 7002) was repressed under copper stress, conversely improved the transcription level in polar yeast cell. Elongation factors (EF1A, EF1B, and EF‐2) are fundamental regulatory proteins of the translational elongation step in eukaryotic organisms. Elongation factor 1 (EF‐1) is ubiquitous and conserved in function, assisting the elongation part in translation cycle of eukaryotes by the regeneration of GTP (Karring et al., 2002). EF‐1 has also been reported to be overexpressed in *Amaranthus hybridus* L. roots upon exposure to cadmium (Jin et al., 2016). In this experiment, four protein spots, identified as elongation factor EF‐1 alpha subunit (spot 302, 304, 507 and 5614), were upregulated by excess Cu except for spot 304. The upregulation of EF‐1 provoked the gene translation and protein synthesis. The function of ATP‐dependent RNA helicases (ADRH) is implicated in lots of cellular processes including ribosome biogenesis, pre‐mRNA splicing, and translation initiation in the nucleus and mitochondria (Jankowsky, 2011). Additionally, ADRH contributes to the formation of RNA degradosomes and stabilize RNA secondary structure in biology under abiotic stress (Owttrim, 2013). ADRH RhlE (spot 314), one member of DEAD‐box RNA helicase family, was de novo synthesized in Antarctic yeast AN5 under copper stress, that improved the transcription and translation in the nucleus mitochondria, and degraded abnormal RNAs, which was coinciding with the response of *Penicillium janthinellum* under Cu stress (Feng et al., 2017). The inducement of ADRH under copper stress had a specific role in potentially improve yeast adaptation to heavy metal. In addition, streptomyces antibiotic regulatory protein (SARP) control secondary metabolism in Streptomyces (Novakova, Rehakova, Kutas, Feckova, & Kormanec, 2011). Under copper stress, SARP protein (spot 8701) disappeared in Antarctic yeast, which indicated that the biosynthesis downregulation of some secondary metabolite might be benefit for saving of energy and substances to meet the high‐energy demand of Cu‐challenged cells.

As we know, ROS is one of the major toxicities exerted by heavy metals in yeast (Jomova & Valko, 2011). They cause severe damage to biological macromolecules by binding lipids, DNA, proteins, etc. (Lledías, Rangel, & Hansberg, 1998). Previous reports found that some antioxidant proteins were increased in yeast *R. mucilaginosa* RCL‐11 stressed by copper (Irazusta et al., 2012). Peroxiredoxin 6, a unique member of the ubiquitous peroxiredoxin family, was recruited to protect cells against oxidative stress via reduction of H_2_O_2_, hydroperoxides, and peroxynitrite, caused by heavy metal (Asuni, Guridi, Sanchez, & Sadowski, 2015). In this study, mitochondrial peroxiredoxin 6 (spot 2002 and 5109) were found significantly enhanced in yeast cells against the heavy metal stress. Although catalase was showed disappeared after copper inducement, the biochemical determination results indicated that antioxidative reagents (carotenoid and glutathione) and enzymes (SOD, POD, CAT and GR) were accumulated, as shown in Figure 6. These active oxygen scavengers, upregulation in copper stress, controlled the generation of ROS under abiotic conditions (Morigasaki, Shimada, Ikner, Yanagida, & Shiozaki, 2008). In polar yeast cells, several antioxidant substances contributed to the heavy metal tolerance by alleviating the ROS damage (Bae & Chen, 2004).

3β‐Hydroxysteroid dehydrogenase/isomerase (3β‐HSD) is an enzyme that catalyzes the biosynthesis of all classes of hormonal steroids. Our study revealed that the synthesis of 3β‐HSD (spot 5202) increased after copper inducement. In different organisms, lots of steroids have been proved to increase the activities of stress‐related enzymes and decrease the metal uptake to adapt various abiotic stresses, such as heavy metals, salinity, and temperature (Bajguz & Hayat, 2009; Kanwar et al., 2012).

In conclusion, a yeast strain isolated from Antarctic sea‐ice sample and identified as *R. mucilaginosa* AN5, has been revealed for the first time to resist heavy metal toxicity. The results suggest greater capability of this sea‐ice isolate for different heavy metal resistance. Common defense strategies of Antarctic yeast to cope with the stress are followed: (a) altered cell surface morphology and growth rate to adapt to heavy metal stress; (b) a higher level of antioxidant reagents to scavenge the excess ROS and protect cellular components against oxidative damages; (c) the upregulated abundance of glycolysis‐ and TCA cycle‐related enzymes to produce the energy to challenge the heavy metal toxicity; (d) synthesis of proteins involved in nucleotide and protein metabolism to regulate the synthesis of nucleotide acid and replication, transcript and translation of DNA to resist copper stress and maintain metabolic balance; (e) increased synthesis of HSPs/molecular chaperones to refold denatured proteins and keep intercellular homeostasis. All of adaptive patterns might work together to act as key roles in heavy metal resistance mechanisms of Antarctic yeast. Cell functioning under Cu^2+^ stress involves a large number of biochemical reactions through many metabolic pathways to ensure a unified system in cells. Thus, the analysis of some copper‐responded proteins might provide new insights to the heavy metal adaptation. Furthermore work will develop to characterize these important proteins and illuminate their roles in the heavy metal response.

## CONFLICTS OF INTEREST

We declare that none of all authors has any conflicts of interest on the content of this article.
