# Supplementary figures and images for: Copper stress response in yeast Rhodotorula mucilaginosa AN5 isolated from sea ice, Antarctic
Source: Microbiologyopen. 2018 Jun 21;8(3):e00657. doi: 10.1002/mbo3.657 (PMC6436437; doi:10.1002/mbo3.657)

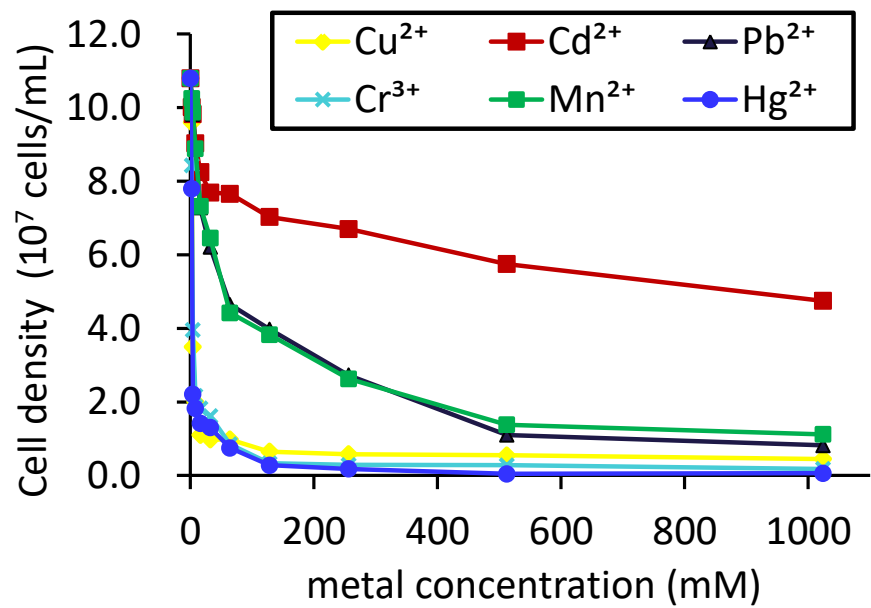

Supplement: Supplementary file 1 [file MBO3-8-e00657-s001.pdf]
